# Parametric Optimization of Electric Discharge Machining of Metal Matrix Composites Using Analytic Hierarchy Process

**DOI:** 10.3390/mi12111289

**Published:** 2021-10-21

**Authors:** Sarabjeet Singh Sidhu, Timur Rizovich Ablyaz, Preetkanwal Singh Bains, Karim Ravilevich Muratov, Evgeny Sergeevich Shlykov, Vladislav Vitalyevich Shiryaev

**Affiliations:** 1 Mechanical Engineering Department, Sardar Beant Singh State University (Formerly Known as Beant College of Engineering and Technology), Gurdaspur 143521, India; sarabjeetsidhu@yahoo.com; 2 Perm National Research Polytechnic University, 614000 Perm, Russia; karimur_80@mail.ru (K.R.M.); kruspert@mail.ru (E.S.S.); vlad2117@gmail.com (V.V.S.); 3 Mechanical Engineering Department, IKG Punjab Technical University, Kapurthala 144603, India; preetbains84@gmail.com

**Keywords:** analytical hierarchy process, residual stress, metal erosion rate, surface roughness

## Abstract

The present study reports on the method used to obtain the reliable outcomes for different responses in electric discharge machining (EDM) of metal matrix composites (MMCs). The analytic hierarchy process (AHP), a multiple criteria decision-making technique, was used to achieve the target outcomes. The process parameters were varied to evaluate their effect on the material erosion rate (*MER*), surface roughness (SR), and residual stresses (σ) following Taguchi’s experimental design. The process parameters, such as the electrode material (Cu, Gr, Cu-Gr), current, pulse duration, and dielectric medium, were selected for the analysis. The residual stresses induced due to the spark pulse temperature gradient between the electrode were of primary concern during machining. The optimum process parameters that affected the responses were selected using AHP to figure out the most suitable conditions for the machining of MMCs.

## 1. Introduction

Composite materials have superior properties, such as high strength; high modulus, low coefficient of thermal expansion; and resistance to fatigue, corrosion, and wear. Due to these prominent properties and their high strength-to-weight ratios, composites are extensively used in numerous advanced engineering applications. Composites with different reinforcements (such as fibers or particles) are being researched widely for their use in several applications, including manufacturing and biomedical industries. The composite materials, usually called metal matrix composites (MMCs), consist of a metal or alloy in the ductile phase to absorb and equally distribute the external load and develop a percolating network to position the reinforced fibers or particles. Alongside this, a brittle constituent, i.e., reinforcement, is embedded in the metal matrix [1,2,3].The combined properties of these constituents in MMCs allow for high strength and fractural properties to be attained, as well as high temperature resistance, making them suitable for applications in the automobile and aviation industries, such as braking systems, piston rods, piston pins, and brake discs [4]. These composites are also used as thermal management solutions for high energy density miniature electronic components, such as microprocessor lids, flip-chip lids, and microwave housing, and can replace high-cost materials such as titanium-based alloy [5,6,7]. With the presence of a soft matrix phase and hard reinforced particles in MMCs, precise machining with conventional methods is challenging in terms of avoiding degradation of the material properties.

Such difficulties can be overcome by adopting newer machining methods that can achieve the desired geometry with minimum damage to the material properties [8]. One such method for machining MMCs is electric discharge machining (EDM), which operates by generating controlled electric sparks to machine composite materials with complex geometries and provides a better surface quality with high dimensional accuracy. In this process, the tool electrode produces its replica in the workpiece material, producing a series of discrete electrical sparks that are generated within the dielectric medium. One of the reasons for the tremendous popularity of this process is its ability to machine complex internal contours, even in hard-to-cut materials, with negligible cutting forces [2,8]; however, in this process, the rapid change in temperature gradient of the machined surface results in sub-surface defects such as cracks, spalling, porosity, residual stresses, and metallurgical transformation [9].

For the effective ED machining of MMCs, a higher value of discharge current and shorter pulse-on time is generally recommended. EDM was highlighted by several researchers in the literature as an effective non-traditional machining technique used for shaping and machining of difficult-to-machine materials such as Al-SiC metal matrix composite [10]. In a similar study, a ZrB_2_-40% Cu composite electrode was reported as an alternative electrode with a better material removal rate and tool wear rate than a conventionally used copper tool [11]; however, the diametric overcut and surface roughness were better with the copper tool than the composite tool electrodes [12].

The output responses, such as the material erosion rate (*MER*), tool wear rate (TWR), and surface roughness (SR), have been studied and widely reported for the EDM process [2,8]; however, one critical factor that significantly affects the machined component’s life is the residual stresses induced while machining. These stresses are quantified using destructive and non-destructive routes [13,14,15]. Non-destructive testing (NDT) X-ray diffraction techniques have been successfully used to evaluate the residual stresses in the materials, and accordingly the effect of the process parameters [16].

Many optimization techniques have been used to analyze the effects of non-conventional machining process parameters on the output responses, such as the *MER*, TWR, SR, and residual stresses. For instance, grey relational analysis has been applied to optimize EDM process parameters on Al-10% SiC composites [17].The multi-regression method was used to establish the relationships between the input and output parameters of the wire EDM process [18].The lexicographic goal programming method was used for optimization of EDM process parameters while machining MMC [19].

So far, the previous studies have mostly reported on the optimization of *MER*, TWR, and SR, but very few studies have focused on optimizing the process parameters in order to minimize residual stresses in MMCs. EDM has been widely used for metals and alloys, although its application on MMCs and analyses of the resulting residual stresses have been limited. As such, the present study aims to establish the best process parameter settings for 65vol% SiC/A356.2 and hybrid 10vol% SiC-5vol% quartz/Al. Three output responses, namely the *MER*, SR, and residual stresses, are optimized using the analytic hierarchy process (AHP). The AHP is a decision-aiding tool that involves specifying a goal, measuring the relative importance (priorities), and choosing the relevant criteria [20,21]. One of the advantages of this tool is that it merges both qualitative and quantitative factors. The tool was formulated to exhibit the way the decision-maker thinks and determines the options based on weighted values. The tangible (objective) and non-tangible (subjective) factors can be efficiently coordinated and can provide reliable findings utilizing simple calculations. The AHP is also validated in various other fields, such as for issues linked to the economy, the stock industry, aircraft manufacture, transportation, and in the construction industry [22].

The objectives of this study are as follows:

- Analyze the influence of the EDM process parameters on the 65vol% SiC/A356.2 (sample I, procured from CPS System, Dallas, TX, USA) and 10vol% SiC-5vol% quartz/Al composites (sample II, produced by a controlled environmental stir casting process);
- Evaluate the outcomes, such as the *MER* (metal erosion rate), SR (surface roughness), and σ (residual stresses), utilizing the L18 Taguchi experimental design and optimizing the process parameters using AHP.

## 2. Material and Methods

### 2.1. Material

Two different variants of particulate-reinforced MMCs were used in this study. The material (65vol% SiC/A356.2 metal matrix composite) used in the study was procured in rectangular plates from CPS, Boston, MA, USA. The other specimen used in this experiment was a hybrid metal matrix composite with 10vol% SiC-5vol% quartz in aluminum, which was prepared using the in-house stir-casting method. The material composition was quantified using optical emission spectroscopy (Make: Arun Technology PolySpek-M spectrometer) as 0.384% Zn, 0.498% Cu, 0.424% Fe, 2.063% Si, and 0.354% Pb, with the balance% as Al.

### 2.2. Method

The experiments were conducted on an OSCARMAX (SD550 ZNC, Taiwan) die-sinking EDM machine using a conventional polarity with the selected tool electrodes. The workpieces were machined in EDM oil as a dielectric fluid and with a suspended powder form of copper (5 g/L) and graphite (5 g/L) in the dielectric medium. To ensure the uniform mixing of the suspended powder, a stirrer pedal fixed at 1400 rpm was used during machining. Three tool electrode materials, namely (i) electrolytic copper, (ii) fined-grained graphite (Poco-EDM 3), and (iii) a copper–graphite composite (50% Cu, Grade 673) were used for the experimental study. The electrodes were machined to a cylindrical shape with a diameter of 18mm.

The responses such as the *MER*, SR, and σ were measured after each experimental trial. The *MER* was measured in terms of weight loss per unit time using a digital weighing machine (Chyo-MJ-300). The surface roughness was measured at three different directions of the machined surface using a Mitutoyo (SJ-400) surface roughness analyzer. The developed residual stresses while machining are quantified by X-ray diffraction technique using a PANalytical’sX’PertPro MPD (Almelo, The Netherlands) diffractometer. The diffractometer used in this study was a horizontal, fixed, laboratory-based system. Table 1 shows the brief experimental conditions for the PANalytical’sX’PertPro X-ray stress analysis.

### 2.3. Experimentation

On the basis of the pilot study, workpiece material, dielectric type, tool electrode material, pulse-on and pulse-off durations, and current, the machining parameters were selected. The other factors, such as the open-circuit voltage (~135 V) and flushing pressure (0.6 kg/cm^2^), were maintained as constant during the experimental study. Table 2 shows the control factors and settings used for the experiments.

Because the chosen factors for the experiments involved a combination of two and three levels, the degree of freedom (dof) for2-level factors was 1 and the doffor3-level factors was 2; hence, the total dof required was 11 (i.e., 1 (one 2-level factor) + 5 × 2 (five 3-level factors) = 11). The mixed-level orthogonal array involving a combination of two- and three-level factors with at least 11 dof was designated experimental design (DOE)L18. This DOE methodology used orthogonally designed arrays that significantly reduced the required number of experimental trials to record the necessary data without compromising the output data quality [23]. L18 signifies the18 distinct orthogonal trial conditions performed randomly to remove any undesirable inclinations in the study. The orthogonal arrays contained the two-level factor in column 1, with the option of assigning three-level factors to the other columns. The conditions of the experimental trials after the assignment of factors to a selected array are listed in Table 3. As can be seen from this design matrix, the first column represents the workpiece materials used in the study; thus, the first nine trials represent 65 vol% SiC/A356.2 MMC, hereafter designated as WP I. The remaining nine trials (trials 10 to 18) represent 10 vol% SiC-5 vol% quartz in aluminum, hereafter referred to as WP II. Other factors are assigned to the remaining columns of the L18 array, as listed in Table 3. The 18 experimental trials with two repetitions are performed as per Taguchi’s design in random order. The mean *MER*, SR, and σ are measured at the end of each trial and are presented in Table 3 under output responses. The *MER* is calculated by the weight difference of the workpiece before and after machining, as given by Equation (1):

(1) $$MER=\frac{\left(w_{i}-w_{f}\right)1000}{T}\mathrm{mg}/\min$$

where Δ*w* if the change in weight, i.e., the weights before and after machining (gm); t is the machining time in minutes.

The SR was measured using a Mitutoyo SJ-400 surface roughness tester in terms of the arithmetic average of the absolute value Ra (µm). Each sample was measured from three locations diametrically on the machined surface and was averaged for investigation.

Residual stresses were estimated with the help of the classical X-ray diffraction procedure. The peak diffracted from the (422) plane was selected to measure the shift at a maximum 2θ angle. The change in d-spacing due to strain in the sample was minimal; hence, the highest possible 2θ angle peak was chosen. The relations of the d-spacing (Δd) with the diffraction peak (Δθ) is given by Equation (2):

(2) $$\frac{\Delta d}{\Delta \theta }=\left(-\right)\left(\Delta \theta \right)\cot\theta$$

The stresses were calculated using the classical sin^2^ψ technique [14,16], with the assumption that the stress state is unidirectional. Equation (3) was used to calculate normal the residual stresses:

(3) $$a^{+}=\frac{1}{2}\;({Є}_{\varphi \psi +}+{Є}_{\varphi \psi -})=\frac{1}{2}\;S_{2}\;\sin^{2}\psi (\sigma_{\varphi })+Є\varphi_{0}{}^{\circ}$$

Here, parameter a^+^ is the average of the lattice strain for positive (Є_φψ+_) and negative (Є_φψ−_) values and ψ is the sample alignment (herein, φ = 0°); 1/2S_2_ = (1 + ν)/E, 1/2S_2_ are the X-ray elastic constants (XEC’s) and their values are represented in Table 4.

Equation (4) can be utilized to estimate the shear residual stress in further studies:

(4) $$a^{-}=\frac{1}{2}\;({Є}_{\varphi \psi +}-{Є}_{\varphi \psi -})=\frac{1}{2}\;S_{2}\;\sin\;(2\psi )(\tau_{\varphi })$$

The sample calibration for the stress test is represented below.

**Figure 1:**
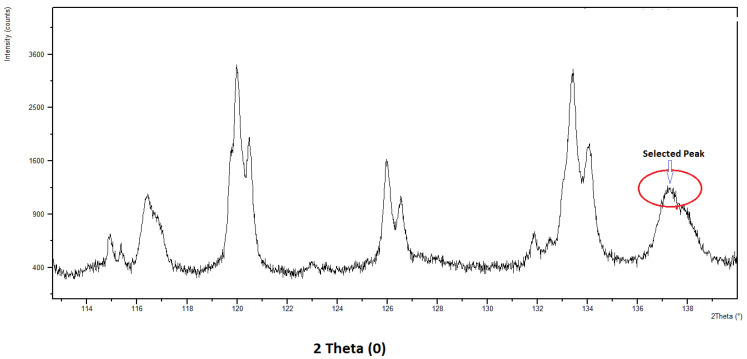
Selected X-ray spectra peaks for residual stress calibration.

*Sample calculation of σ for trial 2 (WP I):* The machined specimen was sectioned to 25 × 25 mm using a wire-cut EDM machine. To limit modifications of the machined surface properties by heating in the wire EDM operation, we ensured that the cutting edge was far away from the calibration surface zone. The etching of re-solidified metal on the machined zone resulted in reduced computation errors. Residual stress was estimated in the aluminum matrix phase of the machined specimen. The measurement was accomplished by selecting the isolated peak diffracted at the highest value of 2θ from the plane. Figure 1 shows the X-ray spectra for trial 2. From the obtained X-ray spectra, the peak selected for residual stress determination was at approximately 137.23°.

Table 5 represents the various parameters for trial 2 used to measure residual stress at different 20 ψ-tilts (positive and negative).

The normal residual stress was analyzed by comparing the linear fit regression equation obtained from the plot of a+ vs. sin2ψ with Equation (3), i.e., the equation obtained was:

$$a^{+}=\;\left(5.21*\sin^{2}\psi =0.291\right)*10^{-4}\;5.21\times 10^{-4}=\frac{1}{2}S_{2\;}\left(\sigma_{\varphi }\right)$$

where 1/2S_2_ = 6.98 T^−1^ Pa, φ = 0

As such, the normal residual stress ($\sigma_{0}$) for trial 2 was 74.6 MPa.

### 2.4. Analysis of Variance for MER, SR, Residual Stress

The *MER*, SR, and σ results were analyzed using analysis of variance (ANOVA). A summary of the ANOVA used for *MER*, SR, and σ is presented in Table 6. The significant parameters were chosen by comparing the F-values with F-critical at a confidence level of 95%. The higher the F-value, the greater the effect of the parameter on the responses.

*MER*: The ANOVA results show that the current and pulse-on contributed significantly to changes in *MER*. Additionally, the variations in the workpiece material had significant effects on the *MER*. On the contrary, the dielectric medium, pulse-off time, and electrode material had no significant effect. It was observed that with increases in pulse-on time and current, the *MER* increases significantly, since increases in the current and pulse-on time increase the spark energy duration; thus, with increased heat input, the temperature increases, resulting in the workpiece’s higher melting or evaporation rate;
*SR*: The surface roughness of the machined surface was significantly influenced by factors such as the powder concentration, current, and pulse-on time. Furthermore, the two materials showed quite different SR values. The roughness increased with increases in current and pulse-on time, whereas the powder in the dielectric medium improved the surface finish. An increase in current or pulse-on time increases the spark energy, which drives the formation of bigger and deeper craters, leading to a rough machined surface. The addition of powder consistently improved the finish of the machined surfaces; the spark becomes more uniform with increased frequency and widens the spark gap. This reduced the magnitude of the impact forces, resulting in small and shallow craters and lowering the surface roughness [24,25];
*Residual Stress*: The ANOVA results show that the pulse off time, powder mixing in the dielectric medium, and current significantly affected the σ. Additionally, the selected MMCs showed different residual stress values for similar parameter settings. It can be seen from the results that the pulse-on time affected the *MER* and SR but had no effect on σ. On the other hand, the pulse-off time hada significant impact on the development of residual stress due to the re-solidification time duration. The presence of suspended particles in the dielectric medium facilitates the easy formation of plasma channels between the electrode and the workpiece, resulting in lower SR and residual stress. The conductivity of suspended particles plays the major role in determining the SR but has no impact on the development of σ.

## 3. Analytical Hierarchical Process

Optimizing the responses independently results in vastly different parametric combinations of the machining process parameters. For example, if *MER* was optimized separately, this would cause some parameters to increase *MER* (the higher *MER* is, the better the function); however, these parameters may not result in decreased SR. The opposite would be true if SR was optimized individually. The identical condition involves residual stresses optimization. To obtain a result that is close to the target, the responses must be optimized together according to their priority. The analytical hierarchy process (AHP) offers one technique that suggested the best combination of parameters to reach or nearly reach the target. The AHP is simply structured and widely used for multiple-goal decision-making techniques and is classified as a decision-making tool for use under conditions of certainty, i.e., the data are obtained deterministically and the tool is designed for situations in which ideas, feelings, and emotions are quantified into a numerical scale [26]. The main steps used in the implementation of the AHP are as follows:

- Define the objective and evaluation criteria and develop the hierarchical structure, with an objective at the top level, the criteria and sub-criteria at the intermediate level, and the available alternatives at the lowest level;
- Form a pair-wise comparison matrix for each level with respect to the higher level and determine the relative importance of the different alternatives with respect to its immediately superior sub-criteria. The comparison is made on a 9-point “fundamental scale of Saaty”, as represented in Table 7.
- Compute the relative weights for the pair-wise comparison matrices using eigenvector methods;
- Judge the scope of inconsistency by using the largest eigenvector. The judgment of the accepted degree of consistency can be checked by means of the consistency ratio (CR) of the consistency index (CI) with the appropriate value of the random index (RI) from Table 8.
- Repeat the above steps for all levels in the hierarchy, with the overall relative value evaluated by the linear addition function.

**Figure 2:**
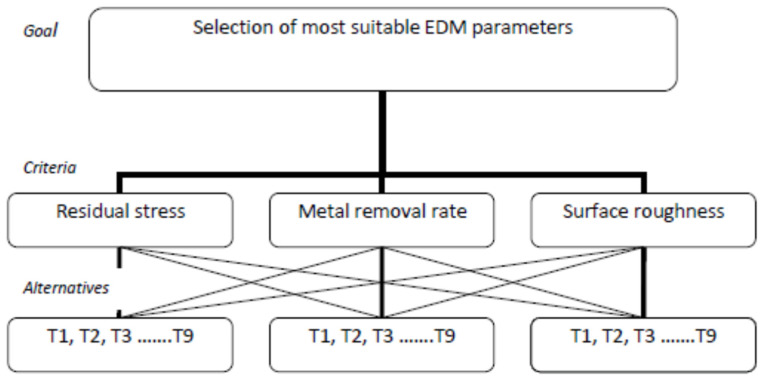
Hierarchy layout of the analytical hierarchy process.

The steps are summarized in Figure 2.

### Implementation of AHP in EDM Process

In the present study, AHP was applied to choose the best combination of input parameters. The criteriawere used to minimize the residual stress and surface roughness and maximize the metal erosion rate. In this experimental design layout, 9 trials are conducted for each type of MMC’s and the orthogonality is maintained by selecting the L18 experimental design. In the present design given in Table 3, trials 1 to 9 are the available alternatives for MMC WP I and trials 10 to 18 are alternatives for MMC WP II. The hierarchy for the AHP in the present study was selected using the steps described above (Figure 2).

The MMCs used in the present study are used for very high-end applications in aerospace and mining, while the residual stresses developed during the EDM process affect the service life of the product. Residual stress was assigned the maximum weight, followed by the material removal rate and surface roughness. To attain the desired objective, the residual stress results were slightly modified (cost-to-benefit conversion, which can be achieved by using the -ve sign), as follows:

(5) $$\sigma_{n}=(\sigma_{\max}-\sigma_{0})+1\;$$

where σ*_max_* is the maximum value of the residual stress in the corresponding trial set for each workpiece, σ_0_ is the residual stress measured with the X-ray diffraction method, and σ_n_ is a modified residual stress value (refer to Table 3), which was calculated from Equation (5).

Using the criteria for assigning weights to the residual stress, *MER*, and SR, a (3 × 1) weight column matrix, as shown in Table 9, was established for pair-wise comparison.

Subsequently a pair-wise comparison of the experimental trials (alternatives) was developed for, σ_n_, *MER*, and SR for each workpiece, with the results shown in Table 10, Table 11, Table 12, Table 13, Table 14 and Table 15. The synthesized matrix to obtain priority vector of σ for WP I is shown in Table 16. It was also ensured during pair-wise comparisons of alternatives that if the values attained during comparison were greater than the maximum limit of Saaty’s fundamental scale, the highest value of the scale (9) was selected to avoid inconsistency.

The pair-wise comparison (9 × 9 matrix) of the alternatives was completed by comparing and rounding off the response ratio obtained in experimental trials. For example, if trial 1 gives a value of 16.5 and trial 2 gives 4.5, then the response ratio is the ratio of the values of the two trials (trial1/trial2), which is 3.66 (rounding off = 4). The same procedure was adopted for all the response parameters to assign the weights. In the work [27] adopted percentage change in the state of tool wear while machining medium carbon steel workpiece. The change in percentage was used to assign the weight in pair-wise comparison matrix.

To illustrate this calculation, the pair-wise matrix for residual stress (σ) (WP I) is considered, as shown in Table 16.

***Step 1:*** The matrix was normalized by dividing each element of the matrix by its column total. For example, for the T(1,1) element, a value of 0.13434 was obtained by dividing 1 by the column total of 7.444 (1 + 1 + 1 + 1 + 1 + 1/3 + 1 + 1 + 1/9). The same procedure was adopted for each element of the matrix, with the results given in Table 16.

***Step 2:*** The estimation of the priority vector was done by taking the row average, i.e., (0.13434 + 0.10405 + 0.10405 + 0.17208 + 0.13433 + 0.12442 + 0.13585 + 0.11843 + 0.12329) and dividing it by 9.

Similarly, the synthesized pair-wise comparison matrix, priority vectors, and validation of the constructed matrix were performed for each response parameter. The overall weight was calculated by multiplying the alternative available priority vectors for each sample with the criteria weight, as given in Table 17.

The overall priority for each EDM parameter setting was calculated as demonstrated below:

*Overall weight of T1 (WP I):* Overall Weight = 0.581552 (0.130829) + 0.308996 (0.02080) + 0.109452 0.0834519) = 0.091645.

Similarly, the overall weight was calculated for each trial conducted for the selected workpieces.

The remaining calculations were completed by combining the assigned criteria weight with the alternative priority weight to obtain the overall priority results (Table 17 and Table 18), as per the hierarchical steps given in Figure 1.

The ideal weight vector was obtained by dividing the priority vector with the largest priority weight element in the matrix. The advantage of using an idealized weight vector is that the ranking of trials does not change due to the influence of a newly introduced non-optimal identical alternative [27].

From the calculated overall priority, the trials were ranked for each type of MMC. The maximum overall weight or composite performance score for sample I was obtained for T4 as given in Table 17 (See also Table 3), which was conducted with a graphite electrode; dielectric medium mixed with Cu powder; pulse-off and pulse-on times of 15 µs and 45 µs, respectively; and current of 8 amps. Similarly, the maximum overall weight or composite performance score for sample II as obtained in Table 18 corresponds to trial number 13 (Table 3) of the original L18 array. This trial was also completed with a graphite electrode; dielectric medium mixed with graphite powder; pulse-off and pulse-on times of 15 µs and 45 µs, respectively; and a current setting of 4 amps. It was observed that the presence of powder in the dielectric medium expanded the area of the spark zone between the electrodes, thereby minimizing the impact of thermal shocks on the machined surface and diminishing the induced residual stresses; thus, the solution that globally optimizes residual stresses, *MER*, and SR for the two types of MMCs (WP I and WP II) used in the experiment was obtained (Table 19).

## 4. Conclusions

In the present study, three output response parameters, namely σ, *MER*, and SR, were optimized using a manageable AHP technique. Due to conflicting parameter settings for different output responses in the EDM process, identifying process parameters is a complex decision-making process. A manageable AHP approach was used in the present study to obtain a more reliable global composite performance score for various trial conditions in powder-mixed electric discharge machining (PMEDM) of MMCs. The process conditions that affected the three responses, namely σ, *MER*, and SR, were identified and optimized using AHP for two different types of MMCs. The current and pulse-on time significantly affected *MER*, while the addition of the powder, current, and pulse-on time influenced SR. Despite this, the pulse-off duration had no significant effect on *MER* or SR. Still, the pulse-off duration had the most considerable influence on residual stresses, followed by the dielectric medium, current, and type of tool electrode.

The three responses were optimized together according to the predetermined goal using AHP. The optimal process conditions for the selected materials were identified. The overall process for both workpieces revealed that machining the workpiece with a graphite tool electrode and higher pulse-on time setting coupled with lowest pulse-off time in the presence of a suspended particle dielectric medium (PMEDM) contributed to minimizing the residual stress with the desired *MER*. Due to the denser ceramic-reinforced particles in WP I compared to WP II, the target results were achieved at a higher current level (i.e., 8A) than the current required for WP II. The optimal settings for achieving the specified target results involved the graphite tool electrode coupled with pulse-on and -off times of 45 µs and 15 µs, respectively, for both workpieces. The methodology used to obtain optimum EDM process parameters can be extended by prioritizing different responses (i.e., *MER*, SR) according to the end-use application of the product. Overall, the use of AHP will open the horizon for EDM practitioners to determinevarious process parameters, improving their ability to achieve their desired targets.

## Figures and Tables

**Table 1:** Residual stress measurement conditions.

Factors	Conditions
Characteristic X-ray	Cu–Kα1 + 2
Measurement method	Ω-Diffractometer method
Diffraction plane, (hkl)	(422)
Tube voltage, KV	45
Tube current, mA	40
Diffraction angle (2θ)	40°–140°
Diffraction plane, (hkl)	(422)

**Table 2:** Parameters with different levels.

Parameters (Symbol)	Levels		
	Level-1	Level-2	Level-3
Workpiece (WP)	65 vol% SiC/A356.2 (WP I)	10 vol% SiC-5 vol% quartz/Al (WP II)	-----
Tool Electrode (TE)	Cu	Gr	Cu-Gr
Dielectric medium(D)	EDM oil (D)	PMEDM (Cu)	PMEDM (Gr)
Current (A) Amp	4	8	12
Pulse-on (T_on_) µs	10	30	50
Pulse-off (T_off_) µs	15	30	45

**Table 3:** Experimental layout (L18).

Trial. No. (T(n)) Where n = 1–18	Process Parameters						Output Responses			
	WP	TE	T_off_ (µs)	T_on_ (µs)	D	A	σ_0_ (MPa)	σ_n_ (MPa) Calculated from Equation (5)	*MER* (mg/min)	SR (µm)
1	WP I	1	15	10	1	4	63.3	66.7	2.64	2.94
2	WP I	1	30	30	2	8	74.6	55.4	14.275	2.05
3	WP I	1	45	45	3	12	82.8	47.2	23.17	5.67
4	WP I	2	15	45	2	8	36.3	93.7	23.38	2.09
5	WP I	2	30	10	3	12	63.6	66.4	18.97	4.12
6	WP I	2	45	30	1	4	110.3	19.7	3.04	3.00
7	WP I	3	15	30	1	12	61.4	68.6	22.240	5.01
8	WP I	3	30	45	2	4	78.5	51.5	9.860	2.06
9	WP I	3	45	10	3	8	129	1	9.460	5.06
10	WP II	1	15	30	3	8	70.4	162.1	20.90	6.69
11	WP II	1	30	45	1	12	104	128.5	60.67	10.46
12	WP II	1	45	10	2	4	78.1	154.4	10.860	4.69
13	WP II	2	15	45	3	4	41.8	190.7	57.99	6.46
14	WP II	2	30	10	1	8	149.3	83.2	18.86	8.44
15	WP II	2	45	30	2	12	132.9	99.6	29.96	4.44
16	WP II	3	15	10	2	12	77.7	154.8	65.5	6.76
17	WP II	3	30	30	3	4	89.2	143.3	10.07	6.12
18	WP II	3	45	45	1	8	231.5	1	45.72	7.95

**Table 4:** X-ray elastic constants.

	Sample I	Sample II
**Elastic constants T^−1^ Pa (1/2S_2_)**	6.98	16.84

**Table 5:** X-ray spectra of lattice strain intrial 2.

	ψ	Sin^2^ ψ	d_φψ_	Є_ψφ_	a+	a−
	0	0	0.827269	0		
**Positive ψ**	12.92	0.05	0.827286	0.00002050	5.08 × 10^−5^	−3.02 × 10^−5^
	18.44	0.1	0.827311	0.00005060	−1.70 × 10^−5^	6.76 × 10^−5^
	22.79	0.15	0.827472	0.00024500	1.97 × 10^−4^	4.82 × 10^−5^
	26.57	0.2	0.827425	0.00018900	2.48 × 10^−4^	−5.90 × 10^−5^
	30.00	0.25	0.827450	0.00021900	1.84 × 10^−4^	3.52 × 10^−5^
	33.21	0.3	0.827499	0.00027800	9.01 × 10^−5^	1.88 × 10^−4^
	36.27	0.35	0.827564	0.00035700	2.10 × 10^−4^	1.47 × 10^−4^
	39.23	0.4	0.827434	0.00020000	1.11 × 10^−4^	8.91 × 10^−5^
	42.13	0.45	0.827673	0.00048800	2.94 × 10^−4^	1.94 × 10^−4^
	45	0.5	0.827543	0.00033100	3.58 × 10^−4^	−2.73 × 10^−5^
**Negative ψ**	12.92	0.05	0.827336	0.00008099	$a^{+}=\frac{1}{2}\;({Є}_{\varphi \psi +}+{Є}_{\varphi \psi -})\;(\mathrm{for}\mathrm{normal}\mathrm{stress})\;\;a^{-}=\frac{1}{2}\;({Є}_{\varphi \psi +}-{Є}_{\varphi \psi -})\;(\mathrm{for}\mathrm{shear}\mathrm{stress})\;\mathrm{where}\varphi =0$	
	18.44	0.1	0.827199	−0.00008462		
	22.79	0.15	0.827392	0.00014868		
	26.57	0.2	0.827523	0.00030703		
	30.00	0.25	0.827392	0.00014868		
	33.21	0.3	0.827188	−0.00009791		
	36.27	0.35	0.827321	0.00006286		
	39.23	0.4	0.827287	0.00002176		
	42.13	0.45	0.827351	0.00009912		
	45	0.5	0.827588	0.00038561		

**Table 6:** Analysis of variance for *MER*, SR, and σ.

Factors	dof	Sum of Squares			Variance			F-Value		
		*MER*	SR	σ	*MER*	SR	σ	*MER*	SR	σ
**W/Pc**	1	1977.26	50.0333	4204.4	1977.26	50.0333	4204.4	10.50 *	80.99 *	9.13 *
**Electrode**	2	62.50	1.9590	3284.0	31.25	0.9795	1641.98	0.17	1.59	3.57 *
**Pulse-off**	2	435.50	0.9768	14262.5	217.75	0.4884	7131.27	1.16	0.79	15.49 *
**Pulse-on**	2	1448.53	4.6520	110.5	724.26	2.3260	55.26	3.85 *	3.77 *	0.12
**Dielectric medium**	2	10.67	22.5037	6526.1	5.33	11.2519	3263.04	0.03	18.21 *	7.09 *
**Current**	2	1494.86	10.6571	4725.5	747.43	5.3286	2362.75	3.97 *	8.63 *	5.13 *
**Error**	6	1129.73	3.7064	2762.6	188.29	0.6177	460.44			
**Total**	17	6559.04	94.4886	35875.7						

* Significant factor.

**Table 7:** Saaty’s fundamental scale.

Scale Value	Explanation
1	Equally preferred
3	Slightly more preferred
5	Strongly preferred
7	Very strongly preferred
9	Extremely preferred
2, 4, 6, 8	Used to reflect compromise between scale values

**Table 8:** Random consistency index.

*k*	1	2	3	4	5	6	7	8	9	10	11	12	13
RI	0.00	0.00	0.58	0.90	1.12	1.24	1.32	1.41	1.45	1.49	1.51	1.48	1.48

**Table 9:** AHP pair-wise comparison of weighting criteria.

	σ	*MER*	SR	Priority Vector
**σ**	1	2	5	0.581552
***MER***	1/2	1	3	0.308996
**SR**	1/5	1/3	1	0.109452
	λ_max_ = 3.00369	CI = 0.0018473, RI = 0.58, CR = 0.003		

**Table 10:** Pair-wise comparison of σ values against their alternatives for WP I.

.	T1	T2	T3	T4	T5	T6	T7	T8	T9	Priority Vector
**T1**	1	1	1	1	1	3	1	1	9	0.130829
**T2**	1	1	1	1/2	1	3	1	1	9	0.12105
**T3**	1	1	1	1/2	1	2	1	1	9	0.115577
**T4**	1	2	2	1	1	5	1	2	9	0.180525
**T5**	1	1	1	1	1	3	1	1	9	0.130829
**T6**	1/3	1/3	1/2	1/5	1/3	1	1/4	1/3	9	0.050517
**T7**	1	1	2	1	1	4	1	1	9	0.136302
**T8**	1	1	1	1/2	1	3	1	1	9	0.12105
**T9**	1/9	1/9	1/9	1/9	1/9	1/9	1/9	1/9	1	0.0133204
	λ_max_ = 9.23034				CI = 0.0287927, RI = 1.45, CR = 0.0198					

**Table 11:** Pair-wise comparison of *MER* values with respect to their alternatives for WP I.

	T1	T2	T3	T4	T5	T6	T7	T8	T9	Priority Vector
**T1**	1	1/5	1/9	1/9	1/7	1	1/8	1/4	1/4	0.02080
**T2**	5	1	1/2	1/2	1	5	1/2	1	1	0.09982
**T3**	9	2	1	1	1	9	1	3	3	0.19547
**T4**	9	2	1	1	1	8	1	2	2	0.17520
**T5**	7	1	1	1	1	6	1	2	2	0.15448
**T6**	1	1/5	1/9	1/8	1/6	1	1/7	1/3	1/3	0.02328
**T7**	8	2	1	1	1	7	1	2	2	0.17034
**T8**	4	1	1/3	1/2	1/2	3	1/2	1	1	0.08303
**T9**	4	1	1/3	1/2	1/2	3	1/2	1	1	0.08303
	λ_max_ = 9.02762				CI = 0.00640, RI = 1.45, CR = 0.0023					

**Table 12:** Pair-wise comparison of SR values with respect to their alternatives for WP I.

	T1	T2	T3	T4	T5	T6	T7	T8	T9	Priority Vector
**T1**	1	1	1/2	1	1	1	1/2	1	1/2	0.0834519
**T2**	1	1	1/3	1	1/2	1	1/2	1	1/2	0.0728733
**T3**	2	3	1	3	1	2	1	3	1	0.176214
**T4**	1	1	1/3	1	1/2	1	1/2	1	1/2	0.0728733
**T5**	1	2	1	2	1	1	1	2	1	0.133865
**T6**	1	1	1/2	1	1	1	1/2	1	1/2	0.0834519
**T7**	2	2	1	2	1	1	1	2	1	0.152199
**T8**	1	1	1/3	1	1/2	1/2	1	1	1/2	0.0728733
**T9**	2	2	1	2	1	1	1/2	2	1	0.152199
	λ_max_ = 9.10338				CI = 0.0129221, RI = 1.45, CR = 0.008911					

**Table 13:** Pair-wise comparison of σ values with respect to their alternatives for WP II.

	T10	T11	T12	T13	T14	T15	T16	T17	T18	Priority Vector
**T10**	1	1	1	1	2	2	1	1	9	0.138612
**T11**	1	1	1	1/2	2	1	1	1	9	0.119842
**T12**	1	1	1	1	2	2	1	1	9	0.138612
**T13**	1	2	1	1	2	2	1	1	9	0.165782
**T14**	1/2	1/2	1/2	1/2	1	1	1/2	1/2	9	0.0758749
**T15**	1/2	1	1/2	1/2	1	1	1/2	1/2	9	0.089421
**T16**	1	1	1	1	2	2	1	1	9	0.129583
**T17**	1	1	1	1	2	2	1	1	9	0.128872
**T18**	1/9	1/9	1/9	1/9	1/9	1/9	1/9	1/9	1	0.0134007
	λ_max_ = 9.1803				CI = 0.0225376, RI = 1.45, CR = 0.0155					

**Table 14:** Pair-wise comparison of *MER* values with respect to their alternatives for WP II.

	T10	T11	T12	T13	T14	T15	T16	T17	T18	Priority Vector
**T10**	1	1/3	2	1/3	1	1	1/3	2	1/2	0.068095
**T11**	3	1	6	1	3	2	1	6	1	0.185247
**T12**	1/2	1/2	1	1/5	1/2	1/3	1/6	1	1/4	0.0330161
**T13**	3	3	3	1	3	2	1	6	1	0.181600
**T14**	1	1	1	1	1	1/2	1/3	2	1/2	0.0629946
**T15**	1	1	1	1	1	1	1/2	3	1/2	0.0923417
**T16**	3	3	3	3	3	3	1	6	1	0.1852470
**T17**	1/2	1/2	1/2	1/2	1/2	1/2	1/2	1	1/5	0.0314636
**T18**	2	2	2	2	2	2	2	2	1	0.159995
	λ_max_ = 9.05235				CI = 0.00654334, RI = 1.45, CR = 0.00451					

**Table 15:** Pair-wise comparison of SR values with respect to their alternatives for WP II.

	T10	T11	T12	T13	T14	T15	T16	T17	T18	Priority Vector
**T10**	1	1/2	1	1	1	2	1	1	1	0.107181
**T11**	2	1	2	2	1	2	2	2	1	0.171361
**T12**	1	1/2	1	1	1/2	1	1	1	1/2	0.0858196
**T13**	1	1/2	1	1	1	1	1	1	1	0.0990902
**T14**	1	1	2	1	1	2	1	1	1	0.126548
**T15**	1/2	1/2	1	1	1/2	1	1/2	1	1/2	0.0741207
**T16**	1	1/2	1	1	1	2	1	1	1	0.107181
**T17**	1	1/2	1	1	1	1	1	1	1	0.0990902
**T18**	1	1	2	1	1	2	2	1	1	0.12661
	λ_max_ = 9.16155				CI = 0.0201939, RI = 1.45, CR = 0.0139					

**Table 16:** Synthesized matrix of σ for WP I.

	T1	T2	T3	T4	T5	T6	T7	T8	T9
**T1**	0.13434	0.10405	0.10405	0.17208	0.13433	0.12442	0.13585	0.11843	0.12329
**T2**	0.13434	0.10405	0.10405	0.08604	0.13433	0.12442	0.13585	0.11843	0.12329
**T3**	0.13434	0.10405	0.10405	0.08604	0.13433	0.08295	0.13585	0.11843	0.12329
**T4**	0.13434	0.20809	0.20809	0.17208	0.13433	0.20737	0.13585	0.23685	0.12329
**T5**	0.13434	0.10405	0.10405	0.17208	0.13433	0.12442	0.13585	0.11843	0.12329
**T6**	0.04478	0.05202	0.05202	0.03442	0.04478	0.04147	0.03396	0.03948	0.12329
**T7**	0.13434	0.20809	0.20809	0.17208	0.13433	0.16590	0.13585	0.11843	0.12329
**T8**	0.13434	0.10405	0.10405	0.08604	0.13433	0.12442	0.13585	0.11843	0.12329
**T9**	0.01493	0.01156	0.01156	0.01912	0.01493	0.00461	0.01509	0.01316	0.01370

**Table 17:** Overall weight matrix of WP I for the priority vector.

Trials	σ (0.581552)	*MER* (0.308996)	SR (0.109452)	Overall Priority Vector	Ideal Weight Vector
T1	0.130829	0.02080	0.0834519	0.091645	0.548453
T2	0.12105	0.09982	0.0728733	0.109219	0.653623
T3	0.115577	0.19547	0.176214	0.146899	0.879124
**T4**	**0.180525**	**0.17520**	**0.0728733**	**0.167097**	1.000000 *
T5	0.130829	0.15448	0.133865	0.138471	0.828682
T6	0.050517	0.02328	0.0834519	0.045707	0.273534
T7	0.136302	0.17034	0.152199	0.148560	0.889066 **
T8	0.12105	0.08303	0.0728733	0.103185	0.617514
T9	0.0133204	0.08303	0.152199	0.049217	0.294540

* 1st rank, ** 2nd rank.

**Table 18:** Overall weight matrix of WPII for the priority vector.

Trials	σ (0.581552)	*MER* (0.308996)	SR (0.109452)	Overall Priority Vector	Ideal Weight Vector
T10	0.138612	0.068095	0.107181	0.113791	0.73091
T11	0.119842	0.185247	0.171361	0.146440	0.940701
T12	0.138612	0.0330161	0.0858196	0.100555	0.645949
**T13**	**0.165782**	**0.181600**	**0.0990902**	**0.155672**	1.000000 *
T14	0.0758749	0.0629946	0.126548	0.077638	0.498728
T15	0.089421	0.0923417	0.0741207	0.088929	0.571260
T16	0.129583	0.1852470	0.107181	0.149991	0.963508 **
T17	0.128872	0.0314636	0.0990902	0.095874	0.615871
T18	0.0134007	0.159995	0.12661	0.071110	0.456796

* 1st rank, ** 2nd rank.

**Table 19:** Summarized process parameters for the target responses.

Parameter	WP I	WP II
Tool Electrode	Graphite	Graphite
Dielectric medium	PMEDM (Cu)	PMEDM (Gr)
Pulse-off time	15 µs	15 µs
Pulse-on time	45 µs	45 µs
Current	8 Amp	4 Amp
